# Integrated Multiscale Multilevel Approach to Open Shell Molecular Systems

**DOI:** 10.1021/acs.jctc.2c00805

**Published:** 2023-02-13

**Authors:** Tommaso Giovannini, Gioia Marrazzini, Marco Scavino, Henrik Koch, Chiara Cappelli

**Affiliations:** † Scuola Normale Superiore, Piazza dei Cavalieri 7, 56126 Pisa, Italy; ‡ Department of Chemistry, Norwegian University of Science and Technology, 7491 Trondheim, Norway

## Abstract

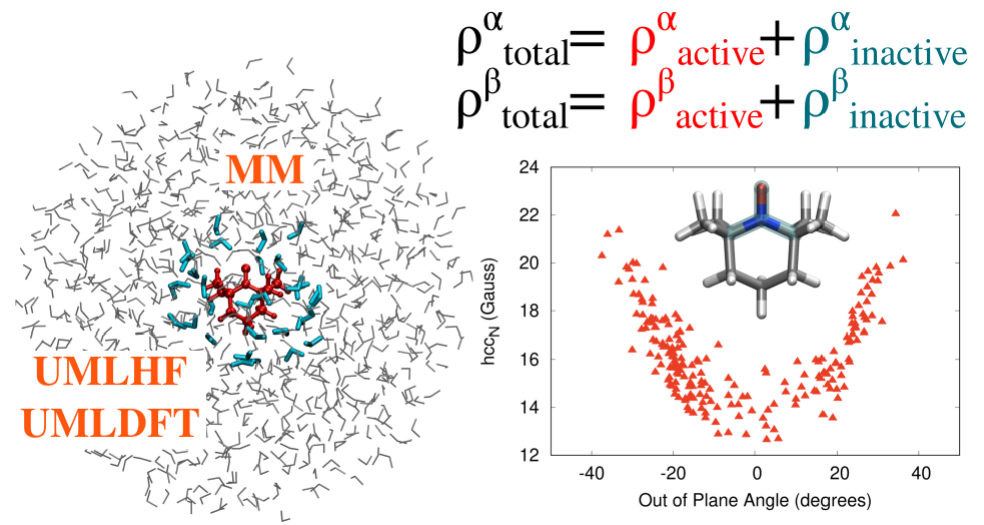

We present a novel multiscale approach to study the electronic structure of open shell molecular systems embedded in an external environment. The method is based on the coupling of multilevel Hartree–Fock (MLHF) and Density Functional Theory (MLDFT), suitably extended to the unrestricted formalism, to Molecular Mechanics (MM) force fields (FF). Within the ML region, the system is divided into active and inactive parts, thus describing the most relevant interactions (electrostatic, polarization, and Pauli repulsion) at the quantum level. The surrounding MM part, which is formulated in terms of nonpolarizable or polarizable FFs, permits a physically consistent treatment of long-range electrostatics and polarization effects. The approach is extended to the calculation of hyperfine coupling constants and applied to selected nitroxyl radicals in an aqueous solution.

## 1 Introduction

The theoretical description of large molecular systems in the condensed phase at a high level of accuracy is challenging, due to the substantial number of degrees of freedom (electronic and nuclear) that need to be treated computationally. However, the complexity can be drastically reduced by partitioning the total system into smaller, interacting subsystems.^1−3^ Solvated molecules, drugs interacting with biological matrices (e.g., DNA and proteins), or molecular systems adsorbed on metal surfaces are generally tackled in this way,^4−8^ by resorting to “focused” computational approaches. There, the total system is partitioned into layers, which are treated at a different degree of sophistication.

The most used focused approaches are defined in the framework of QM/classical methods, where the attention is focused on the QM layer, whereas the rest of the system is described in terms of classical physics. Generally, the atomistic nature of the whole system is retained, such as in QM/molecular mechanics (QM/MM) approaches.^8,9^ The interaction between the QM and classical moieties is modeled in terms of classical electrostatics, and in some cases, mutual polarization effects are considered.^5^ Only a few examples exist, where purely QM interactions, such as Pauli repulsion and dispersion forces, are coherently introduced in the QM/classical modeling,^10−13^ even though they play a crucial role in many systems.^14,15^ Finally, the quality of QM/classical methods also depends on the quality of the MM description, which is determined by the availability and reliability of parameter sets.^16^

As an alternative to QM/classical methods, quantum embedding approaches can be exploited.^17−28^ There, both subsystems are described quantum-mechanically, generally at different levels of accuracy. The advantage of quantum vs classical embedding is 2-fold: (i) target-environment interactions are treated at the QM level, and (ii) a full QM description does not require any parametrization; therefore, quantum embedding approaches can potentially be applied to any kind of interacting systems at the same level of accuracy. The price to pay consists of a generally larger computational cost of quantum vs classical embedding, that may limit, even substantially, the size of actually treatable systems. For this reason, the development of computationally effective yet physically consistent approaches is mandatory. The recently proposed Multilevel HF (MLHF)^29^ and Multilevel DFT (MLDFT)^30^ approaches, which are coherently rooted in the context of Hartree–Fock (HF) and Density Functional Theory (DFT), are a remarkable example of this class of methods.^29−33^ MLHF and MLDFT lie on common theoretical foundations, being based on a decomposition of the total density matrix into active and inactive contributions.^29,30^ There, HF or DFT equations are reformulated in terms of active and inactive density matrices, and the molecular orbitals (MO) coefficients of the active part only are optimized in the Self Consistent Field (SCF) procedure. The inactive density matrix is kept fixed and gives rise to an effective field, which interacts with the active part (See refs (29) and (30) for more details.).

The partitioning of the system that is featured in MLHF/DFT allows substantial extension of the size of chemical systems that may be afforded by HF and especially DFT. However, this gain in size may not be sufficient to treat realistic systems in the condensed phase, i.e. surrounded by an external environment. To this end, quantum embedding approaches may benefit from the coupling with an outer layer described in terms of classical physics, e.g. by means of MM force fields.^34,35^ Remarkably, the coupling is physically grounded, because long-range interactions are dominated by electrostatics and polarization (and dispersion, which is however described neither by HF nor by DFT). Such forces can effectively be described at low computational cost by polarizable MM force fields. In the resulting approach, electrostatics, polarization, and Pauli repulsion interactions are accurately described at the QM level within the MLHF/MLDFT region, whereas long-range interactions are effectively taken into account by means of a polarizable MM level.^34^ As a consequence, the novel class of methods gains advantage from the physicochemical features of both approaches.

In this paper, we integrate classical MM force fields,^8,9^ either within the electrostatic or polarizable embedding schemes (the latter based on the Fluctuating Charge (FQ) force field^4−6,36,37^), with a novel class of multilevel approaches, which extend MLHF and MLDFT to open shell systems. They are based on a unrestricted formulation, which exploits a partition of both α and β densities in active and inactive contributions. Therefore, the computational saving with respect to full HF or DFT descriptions is achieved by keeping frozen the inactive α and β density matrices, which only perturb active densities. The resulting unrestricted MLHF (UMLHF) and UMLDFT are able to account for electrostatic (polarization) forces and Pauli repulsion between active and inactive QM regions, whereas long-range electrostatic and polarization terms are effectively taken into account at a low computational cost through the interaction with the FQ layer. To show the potentialities of the resulting UMLHF(UMLDFT)/MM(FQ) method, it is challenged to compute hyperfine coupling constants (hccs) of selected molecular spin probes.^38−43^ Since hccs are particularly sensitive to the probes’ external environment,^44−46^ they represent an ideal test bed for the novel multiscale multilevel approach.

The manuscript is organized as follows. In the next section, we report the theoretical derivation of UMLHF(UMLDFT)/MM(FQ). The method is then applied to simulate the hcc of the nitrogen atom of PROXYL and TEMPO radicals in an aqueous solution, by exploiting a hierarchy of classical embedding approaches. A summary and a discussion of the future perspective of this work end the manuscript.

## 2 Theory

In this section, the fundamentals of UMLHF(UMLDFT)/MM(FQ) are reported. We first focus on the extension of MLHF/MLDFT to the unrestricted case, thus defining UMLHF and UMLDFT. Then, the way they are coupled with an outer classical, atomistic layer is detailed, by specifying the method either within the electrostatic or polarizable embedding scheme. In the latter case, the coupling with the FQ force field is discussed. Last, the approach is developed for the calculation of hyperfine coupling constants.

### 2.1 Unrestricted MLHF and MLDFT

The starting point to derive UMLHF/UMLDFT is the expression of the energy *E*[**D**^α^,**D**^β^] for open shell systems in the unrestricted formalism
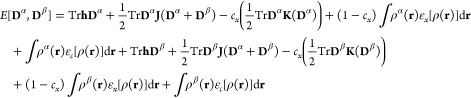
1where **D**^σ^ is α- and β-density matrices (with σ = α, β), **h** is the one-electron operator, and **J** and **K** are Coulomb and exchange matrices, respectively. Eq 1 is formulated for a generic DFT functional, where ρ^σ^(**r**) is α- and β-DFT density functions, ε_*x*_ and ε_*c*_ indicate exchange and correlation energy densities per unit particle, and *c*_*x*_ is the percentage of HF exact exchange. The UHF equations can easily be recovered by imposing *c*_*x*_ = 1 and ε_*c*_ = 0. The total density matrix **D** can be obtained from α- and β-density matrices as **D** = **D**^α^ + **D**^β^.

Similar to the closed shell case,^29,30^ unrestricted MLHF/MLDFT are formulated by separating the total system into active (*A*) and inactive (*B*) parts. From a mathematical point of view, the separation is performed by decomposing α and β density matrices into active **D**_*A*_^σ^ and inactive **D**_*B*_^σ^ contributions:
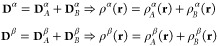
2

A similar partitioning applies to DFT density functions (ρ^α^(**r**), ρ^β^(**r**)). Note that in general **D**_*X*_^α^ ≠ **D**_*X*_^β^, {X = *A*,*B*}. By substituting eq 2 into eq 1, we obtain
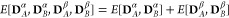
3where *E*[**D**_*A*_^σ^, **D**_*B*_^σ^] ({σ = α, β}) is given by
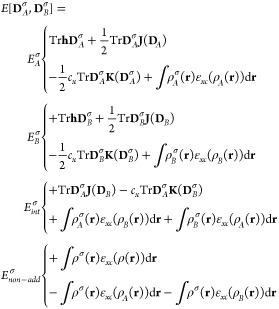
4where the identity Tr**D**_*A*_**G**(**D**_*B*_) = Tr**D**_*B*_**G**(**D**_*A*_) is used.^47^ In eq 4, ε_*c*_ + (1 – *c*_*x*_)ε_*x*_ is substituted by ε_*xc*_ to make the notation compact, and energy terms are separated into active and inactive contributions (*E*_*A*,*B*_^σ^). Note that within this notation, the dependency of *E*[**D**_*A*_^α^, **D**_*B*_^α^] on β densities, through the exchange-correlation functional and the Coulomb matrix, is not explicitly stated. Also, the coupling terms are divided into a purely interaction energy term, *E*_*int*_^σ^, and a nonadditive contribution *E*_*non*–*add*_^σ^, which originates from the nonlinearity of ε_*x*_ and ε_*c*_. It is worth noting that the nonadditive energy terms vanish for UMLHF. As expected, the partitioning of the different terms in eq 4 is coherent with the MLDFT formulation for closed shell systems (See ref (30).). Indeed, as we have previously reported in ref (30), both MLHF(DFT) and their unrestricted formulations differ from other quantum-embedding approaches proposed in the literature. In fact, the partitioning is performed on the density matrix **D** and not on the density function ρ(**r**), as in frozen density embedding (FDE) and embedded density functional theory approaches.^24,48−53^ In this way, it is not necessary to resort to an ad hoc definition of nonadditive kinetic potential terms, either approximated or exact.^20,21,25,54,55^ In MLDFT, the total density matrix is approximated and then partitioned (See eq 2.), thus avoiding the DFT calculation on the whole system. Furthermore, the active density (matrix) is optimized in the field of the inactive density (matrix), which is kept frozen.

As already discussed in the Introduction, the energy of the active fragment *A* is optimized, while the inactive density matrix *B* is kept fixed to the value resulting from the partitioning in eq 2. Therefore, the UMLHF/MLDFT α and β Fock matrix (*F*_*μν*_^σ^, in the AO basis {χ_μ_}) can easily be recovered by differentiating the energy in eq 4 with respect to the active density matrix (*D*_*A*_^σ^), i.e.
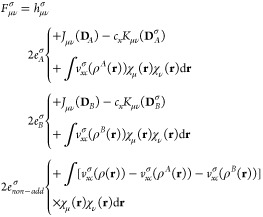
5where we have used the compact notation *v*_*xc*_ = (1–*c*_*x*_)*v*_*x*_ + *v*_*c*_. The two-electron contributions to the Fock matrix can be grouped into different 2*e*_*X*_ terms, with X = *A*, *B*. 2*e*_*non*–*add*_^σ^ is due to the nonlinearity in the DFT functional and again vanishes for UMLHF. Finally, note that 2*e*_*B*_^σ^ accounts for the frozen fragment, of which the density (*D*_*B*_, ρ^*B*^(**r**)) does not change along SCF cycles. Therefore, 2*e*_*B*_^σ^ is a constant one-electron contribution, which is computed only once, at the beginning of the SCF procedure, similar to MLHF/MLDFT.^29,30^

We further point out that UMLHF and UMLDFT equations directly follow from the partitioning of total α and β densities into active and inactive contributions. Similar to their closed shell counterparts, their accuracy depends on the approach which is exploited to carry out the decomposition in eq 2. Different choices are possible; however, in the present work, the initial set of active occupied molecular orbitals (MOs) is obtained through a partial limited Cholesky decomposition of **D**^σ^.^56,57^ In ref (33) the procedure is detailed; however, its extension to open shell systems is not straightforward. In fact, for UMLHF/MLDFT, the Cholesky decomposition needs to be performed twice, i.e. for both **D**_*A*_^σ^ (σ = α, β) densities. **D**_*A*_^σ^ can be written in the AO basis {μ, ν} as follows^58^
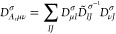
6where
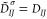
7
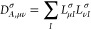
8where the diagonal elements *I* and *J* are decomposed, the **D̃** submatrix contains the selected diagonal elements, and *L*_*αI*_ is the Cholesky orbitals. Diagonal elements are selected so that pivots correspond to the AOs belonging to a predefined set of active atoms. As a result of the decomposition, the active Cholesky occupied MOs are obtained, and the active density matrices **D**_*A*_^σ^ are trivially constructed (See eq 8.). The active virtual space can be defined in terms of projected atomic orbitals (PAOs),^59,60^ which are obtained by projecting out occupied components from the subset of AOs centered on the active atoms. Possible linear dependencies are removed by Löwdin orthonormalization (See also ref (32).).

In the practical implementation, UML calculations follow this protocol:

1. Generation of the guess AO density matrices (**D**^α^ and **D**^β^) by superposition of atomic density (SAD) matrices, obtained through a UHF calculation.
2. Construction of initial Fock matrices from the SAD density matrix and diagonalization, so to obtain initial idempotent α and β densities. At this step, a part of polarization and charge-transfer effects between active and inactive parts is included. Although such terms are introduced by a rather crude approximation of the total density matrix, the numerical results (vide infra) demonstrate that such a choice is indeed able to catch the largest part of these interactions.
3. Partitioning of the density matrices (α and β) into active *A* and inactive *B* densities (**D**_*A*_^α^, **D**_*A*_^β^ and **D**_*B*_^α^, **D**_*B*_^β^). As discussed in the previous section, for occupied orbitals, this step is performed by partial Cholesky decomposition. Virtual orbitals are instead obtained by means of PAOs or by decomposing the virtual density matrix by using the Cholesky algorithm. Notice that the whole procedure generates orthogonal MOs between the active and inactive parts. For the active part, the resulting MOs are used to transform matrices from the AO to the MO basis of the active part only.
4. Calculation of constant energy terms and one-electron contributions due to the α and β density matrices of the inactive part (See eqs 4 and 5.).
5. Energy minimization (eq 4) in the MO basis of the active part only, until convergence is reached.

### 2.2 Coupling with an Outer Layer Described with MM Force Fields

The UMLHF(UMLDFT)/MM(FQ) is defined by starting from the total energy of the system, i.e.
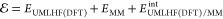
9where *E*_UMLHF(DFT)_ is given in eq 4, whereas *E*_MM_ and *E*_UMLHF(DFT)/MM_^int^ are MM and UMLHF(DFT)/MM interaction energies, respectively. Electrostatic and polarizable QM/MM embeddings differ from the way the interaction energy is specified (in our case *E*_UMLHF(DFT)/MM_^int^); electrostatic embedding approaches limit the description to electrostatic forces only, whereas mutual QM/MM polarization is modeled in polarizable embedding approaches.^5,8^ In particular, nonpolarizable embedding methods place fixed charges on MM atoms, which polarize the QM density. Different polarizable QM/MM approaches exist;^13,61−66^ in this work, we exploit QM/Fluctuating Charges (FQ),^5,6^ where each MM atom is assigned an atomic electronegativity (χ) and chemical hardness (η), which give rise to electric charges (*q*) as a response to the atomic chemical potential.^4,5,67−69^ Therefore, for both nonpolarizable UMLHF(DFT)/MM or polarizable UMLHF(DFT)/FQ, the UMLHF (DFT)/MM interaction energy can be written as follows
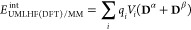
10where *V*_*i*_(**D**^α^ + **D**^β^) is the electric potential generated by the total QM α and β density matrix (i.e., both active and inactive contributions) on the i-*th* charge (*q*_*i*_). In the case of nonpolarizable QM/MM, *q*_*i*_ values are fixed, whereas in QM/FQ, they are obtained by minimizing the following energy expression
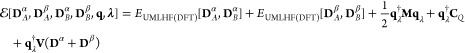
11where *E*_UMLHF(DFT)_[**D**_*A*_^α^, **D**_*B*_^α^] and *E*_UMLHF(DFT)_[**D**_*A*_^β^, **D**_*B*_^β^] are due to the total α- and β densities, respectively (See eq 4.). In eq 11, **q**_λ_ indicates a vector collecting FQ charges and a set of Lagrangian multipliers, which ensure charge conservation on each fragment composing the MM layer (e.g., on each solvent molecule for solvated systems). The **M** matrix is the interaction kernel between the FQ charges, which also contains the Lagrangian blocks,^36^ and the vector **C**_*Q*_ accounts for the interaction between permanent moments, i.e. χ and charge constraints *Q* on each FQ moiety.

The FQ charges equilibrated for the UMLHF(DFT)/FQ systems are obtained by minimizing the energy functional in eq 11. This procedure yields the following set of linear equations
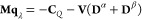
12In parallel, UMLHF(DFT)/MM α- and β-Fock matrices are defined as follows
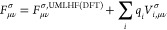
13where *F*_*μν*_^σ,UMLHF(DFT)^ is defined in eq 5. As for energy contributions, the additional QM/MM term is fixed and computed only at the first SCF cycle in case of nonpolarizable QM/MM, whereas it changes in QM/FQ, because FQ charges depend on QM densities. Therefore, the UMLHF(DFT)/FQ contribution to the Fock matrix needs to be updated at each SCF cycle, thus introducing mutual polarization effects between UMLHF(DFT) and FQ layers.

### 2.3 Hyperfine Coupling Constants

To highlight the potentialities of UMLHF(DFT)/MM(FQ), in this paper, we focus on hyperfine coupling constants. The hyperfine interaction between electron spin **S** and nuclear spin **I** can be calculated in terms of the hyperfine coupling tensor **A**, which for a given nucleus *X* reads^70^
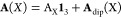
14

In eq 14, the dipolar contribution **A**_dip_(X) is a zero-trace tensor and vanishes in isotropic media (e.g., solutions). The A_X_ denotes the so-called hyperfine coupling constant (hcc), which is connected to the spin density function (ρ_*X*_) at nucleus X through the following equation
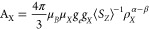
15where μ_*B*_ is the Bohr magneton, and *g*_*e*_ is the free electron g-factor (*g*_*e*_ = 2.0022319); whereas μ_*X*_ and *g*_*X*_ refer to nucleus *X*. The ρ_*X*_^α–β^ reads
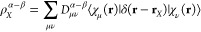
16

*D*^α–β^ is the difference between α and β density matrices. UMLHF(DFT) hccs are computed for the active atoms only, and the difference between α and β density matrices in eq 16 refers to both active and inactive spin-density matrices, which are calculated by minimizing UMLHF(DFT) energy in eqs 4-5. When the outer MM layer is considered, the active spin-densities entering eq 16 are obtained by minimizing the UMLHF(DFT)/MM energy (See eqs 11-13.).

## 3 Computational Details

**Figure 1:**
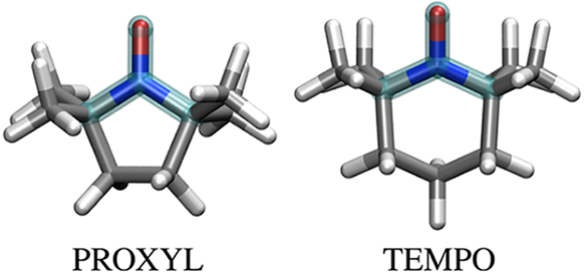
PROXYL (left) and TEMPO (right) molecular structures. The atoms involved in the out-of-plane angle are highlighted in cyan in both radicals.

In the following sections, UMLDFT is applied to the calculation of the hcc_N_ of PROXYL and TEMPO nitroxyl radicals in an aqueous solution (See Figure 1.). Such systems are characterized by the presence of the N–O group, which has been amply exploited as “spin probe” for structural studies of macromolecular systems.^43^ In particular, in order to take into account the dynamical aspects of the solvation phenomenon, we resort to the computational protocol suitably designed by us for the study of aqueous systems.^5^ First, classical MD simulations of the spin labels in water are performed by using AMBER,^71^ according to ref (72) in order to accurately sample the phase-space. From the classical trajectories, 200 uncorrelated snapshots are extracted, and spherical droplets centered on the solutes’ center of mass are cut (radius = 13 Å).

In line with previous studies,^34^ for each snapshot, the radical (TEMPO or PROXYL) and the water shell closer than 3.5 Å with respect to the solute center of mass are described at the UMLDFT level (On average, 30 water molecules are included in the UMLDFT layer.). The remaining water molecules are described classically by means of the polarizable FQ force field, by exploiting the parameters reported in ref (72), specifically developed to correctly describe the electrostatic and polarization interactions of radicals in an aqueous solution. To test the quality of the UMLDFT description, different active/inactive partitions are considered by including 0, 2, 5, or 10 water molecules in the active part (MLDFT_nw_, *n* ∈ [0, 2, 5, 10]). Such molecules are the closest to the radical center of mass (C.M.) or the N–O group (−NO). The remaining water molecules in the UMLDFT region are treated as inactive. The radical is treated at the PBE0/N07D^73^ level, whereas the QM water molecules (either active or inactive) are described at the PBE0/6-31G level. We denote this combination as PBE0/N07D/6-31G(w). Moreover, virtual orbitals are determined by using the PAOs algorithm.

Finally, PROXYL and TEMPO hcc_N_’s are calculated as an average of the 200 uncorrelated snapshots. The structures exploited in this work are available as SI. All QM calculations are performed by using a development version of the electronic structure code *e*^*T*^.^74^

## 4 Numerical Applications

### Testing of the Computational Approach

**Figure 2:**
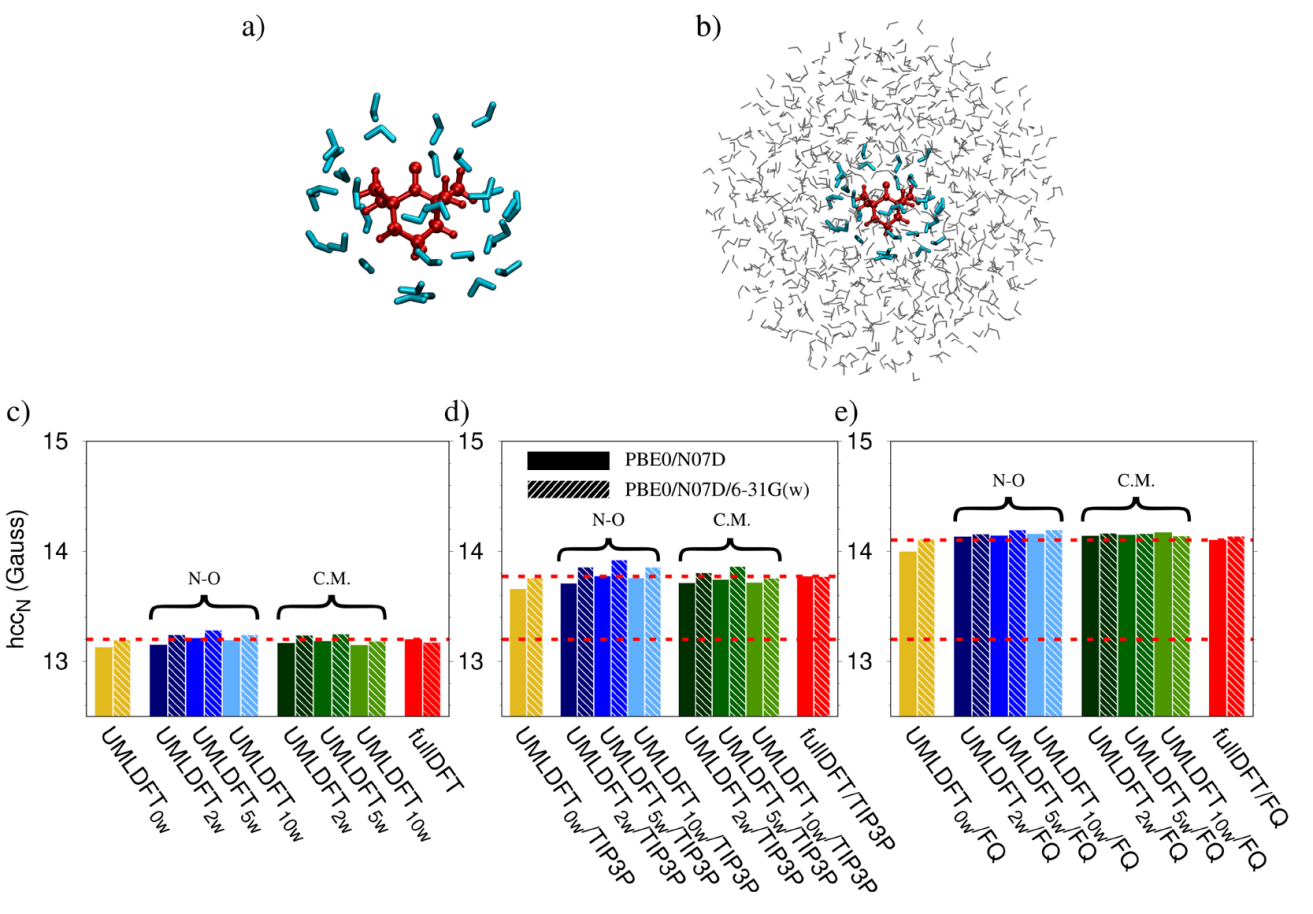
Molecular structure of the randomly selected TEMPO snapshot as described at the UMLDFT (a) and UMLDFT/MM (b) levels. (c-e) hcc_N_ (Gauss) calculated at the UMLDFT_*n*w_(/TIP3P,/FQ) and full DFT(/TIP3P, /FQ) levels. Water molecules included in the UMLDFT layer are selected with respect to the N–O group or C.M. Horizontal red lines correspond to full PBE0/N07D (c-e), PBE0/N07D/TIP3P (d), and PBE0/N07D/FQ (e) results.

To first demonstrate the reliability of the computational approach, a random snapshot is extracted from MD simulations, and hcc_N_’s are computed. To quantify the role of long-range electrostatics interactions, hcc_N_’s are also computed by removing the MM layer (See Figure 2a-b.). The model systems exploited in the analysis are depicted in Figure 2a-b; TEMPO is colored in red, UMLDFT water molecules are colored in cyan, and the MM layer is colored in gray. Additional calculations are performed by exploiting the N07D basis set to describe all of the systems, thus quantifying the effect of polarization and diffuse functions on water molecules, as compared to PBE0/N07D/6-31G(w) calculations (full and patterned boxes in Figure 2c-d). Also, comparison with calculations where the UMLDFT region is treated at the full DFT level is proposed (labeled as fullDFT, fullDFT/TIP3P, and fullDFT/FQ, respectively), that in order to quantify the quality of the ML partitioning. All the results obtained with the different models are summarized in Figure 2 for TEMPO (See also Table 1 for raw data.). The corresponding data for PROXYL are given in Figure S1 in the Supporting Information – SI, together with the associated raw data in Table S1.

**Table 1:**
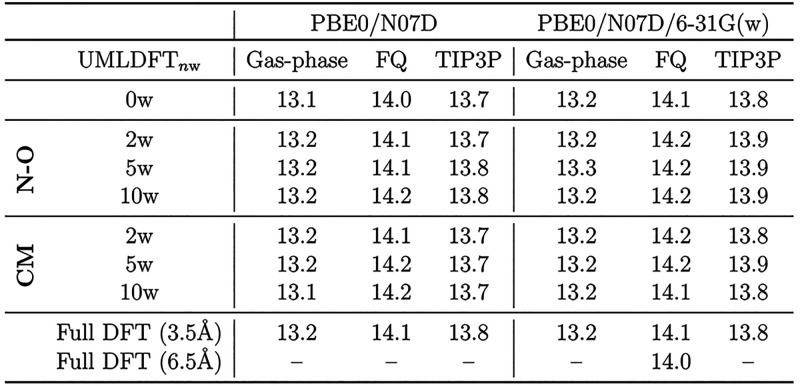
TEMPO hcc_N_ (Gauss) Calculated at the UMLDFT_*n*w_(/TIP3P,/FQ) and Full DFT(/TIP3P, /FQ) Levels for a Randomly Selected Snapshot^a^

a Water molecules included in the UMLDFT layer are selected with respect to the N–O group or C.M.

We first focus on the effect of including additional water molecules in the active fragment. Independently of the basis set exploited, TEMPO and PROXYL hcc_N_’s are not particularly affected by *n*w. In fact, a maximum difference of 0.1 G between 0w and 10w is reported for both radicals, independently of the method used to select the active water molecules (i.e., with respect to the N–O group or the radical C.M.). These data suggest that the most relevant short-range solute–solvent interactions are correctly taken into account by UMLDFT.

Long-range electrostatics (including polarization) plays instead a crucial role, with contributions ranging from 0.85 to 1.3 G when the water molecules are described by means of the polarizable FQ force field (See Figure 2e.). When the nonpolarizable TIP3P force field is instead used, a minor shift of the computed hcc_N_ with respect to the values computed for the small cluster is reported (ranging from 0.5 to 0.6 G, see Figure 2d). The different results provided by QM/TIP3P and QM/FQ can be ascribed to the more realistic description of specific solute–solvent interactions interactions which is provided by the fully polarizable QM/FQ.^5^ Also, the FQ parametrization which is here exploited is tuned to describe electrostatic and polarization energies in an aqueous solution.^72^ As compared to the gas-phase hcc_N_, QM/TIP3P and QM/FQ results clearly show the crucial role of including long-range electrostatic and polarization effects in the description of the hcc_N_ of solvated radical species.

To investigate this point in more detail, TEMPO hcc_N_’s are also computed by increasing the radius of the fullDFT region from 3.5 to 6.5 Å (See Table 1.). A small difference (∼0.1 G) is computed with respect to fullDFT/FQ values obtained by exploiting a cutoff radius of 3.5 Å (See Figure 2.). This further demonstrates that convergence as a function of the size of the fullDFT region is reached and also highlights the better performance of the polarizable FQ with respect to the nonpolarizable TIP3P, for which a larger deviation is reported (See Table 1.). Similar findings are reported for the randomly selected PROXYL snapshot (See Table S1 in the SI.). The data depicted in Figure 2c-e also demonstrate that the water molecules described at the QM level can be treated by using the cheaper 6-31G basis set (i.e., without the need of including polarization and diffuse functions), being the largest difference between full and UML PBE0/N07D or PBE0/N07D/6-31G(w) results is less than 0.1 G.

### 4.1 PROXYL and TEMPO in an Aqueous Solution

On the basis of the benchmarking results reported above, UMLDFT/PBE0/N07D/6-31G(w)/FQ is then applied to the calculation of hcc_N_’s of both PROXYL and TEMPO on the whole set of 200 uncorrelated snapshots extracted from MD runs. Computed UMLDFT_*n*w_/FQ results are reported in Table 2, together with associated standard errors (se) at 67% confidence interval. The latter are computed as
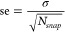
17where σ is the standard deviation for the 200 uncorrelated snapshots (*N*_*snap*_).

**Table 2:**
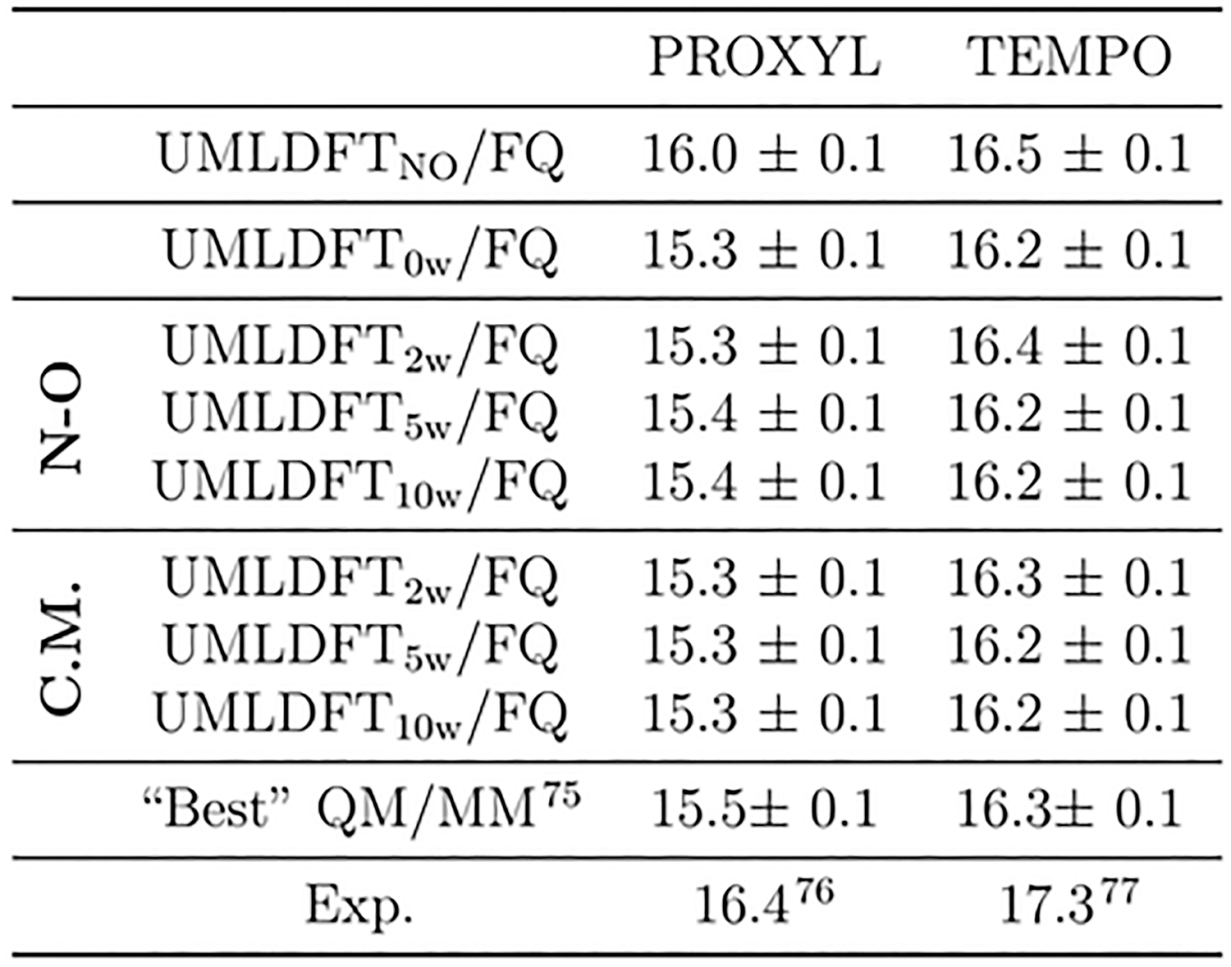
Calculated UMLDFT_*n*w_/FQ hcc_N_ (Gauss) Average Values for PROXYL and TEMPO in an Aqueous Solution^a^

a Values are averaged over 200 uncorrelated snapshots extracted from MD runs. The “best” QM/MM results are reproduced from ref (75), whereas experimental data are taken from refs (76) and (77). Water molecules included in the UMLDFT layer are selected with respect to the N–O group or C.M. In UMLDFT_NO_, the active space is reduced to the N–O group only.

**Figure 3:**
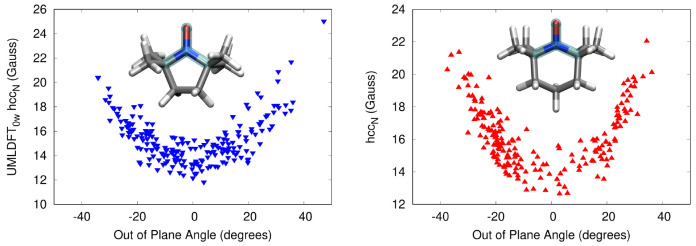
Computed UMLDFT_0w_/FQ hcc_N_ values (Gauss) for PROXYL (left) and TEMPO (right) in an aqueous solution as a function of the out-of-plane angle (See the inset.).

We first note that the inclusion of water molecules in the active region only marginally affects the computed hcc_N_ values, in agreement with the benchmark analysis discussed above for the random snapshot. Interestingly, this result shows that a solvent frozen density, which we remark has been polarized by the solute at the second step of our computational protocol, is able to correctly describe hcc_N_ values. We also investigate the dependence of hcc_N_’s on the out-of-plane dihedral angle involving C–C–N–O atoms, i.e. the nitrogen atom pyrimidalization (See Figure 1.), which has previously been reported to crucially affect the description of hcc_N_’s of the studied radical species.^72,78^ Computed data at the UMLDFT_0w_/FQ level are depicted in Figure 3 for PROXYL (left) and TEMPO (right). The hcc_N_ hugely varies as a function of the OOP angle, ranging from about 12 to 26 G. Note that such a variability is the main reason why 200 snapshots are required to converge the property (See the top panel in Figure 3.) and also demonstrates that a reliable sampling of the radical/solvent phase-space is required to reliably model this property.

**Figure 4:**
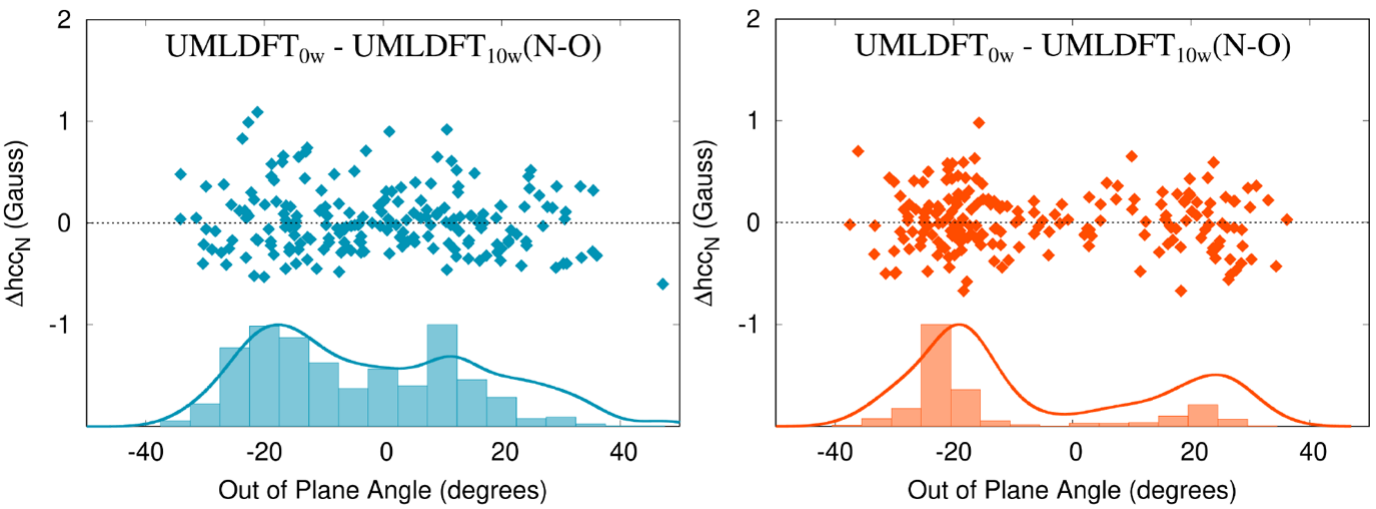
Computed UMLDFT_0w_/FQ - UMLDFT_10w_(N–O) Δhcc_N_ (Gauss) for PROXYL (left) and TEMPO (right) in an aqueous solution as a function of the out-of-plane angle. Histograms and Gaussian convolutions of the absolute Δhcc_N_ values are also given.

The data in Table 2 show that for both radicals, UMLDFT_0w_/FQ and UMLDFT_10w_/FQ provide almost the same computed hcc_N_, independently of how the active water molecules are selected (i.e., with respect to the N–O group or the C.M.). This is in line with previous observations reported in Figure 2. Such results can be further investigated by plotting the difference between hcc_N_ values computed by exploiting the two approaches (Δhcc_N_), as a function of the OOP angle (See Figure 4, where UMLDFT_10w_(N–O) is considered.). For most snapshots, the two approaches give similar hcc_N_ values (Δhcc_N_ = 0 G). However, large differences, ranging from −0.7 to 1.1 G, with a standard deviation of 0.3 G, are reported for specific values of the out-of-plane angle for both PROXYL and TEMPO. Figure 4 also reports absolute values of the error between the two approaches, together with their Gaussian convolution. For TEMPO (left panel of Figure 4), the largest discrepancies are reported for the region near ±20 degrees, and the Gaussian convolution shows a minimum at about 0 degrees. A different situation occurs for PROXYL, for which maxima are located at −20 and 10 degrees. These results show that although average hcc_N_ values computed by UMLDFT_0w_ and UMLDFT_10w_(N–O) are similar for both radical species, a large fluctuation is reported as a function of the snapshot. This confirms that an appropriate sampling of the phase-space is required and that the dynamical aspects of the solvation phenomenon need to accurately be taken into account. In addition, it is worth noting that additional polarization effects which are considered by including the closest water molecules in the active region do not play a crucial role in determining the final average results. However, the present analysis clearly shows that such effects need to be accurately investigated when multilevel methodologies are employed, as already reported by some of us for excitation energies.^34^

### Covalently Bonded Fragments

**Figure 5:**
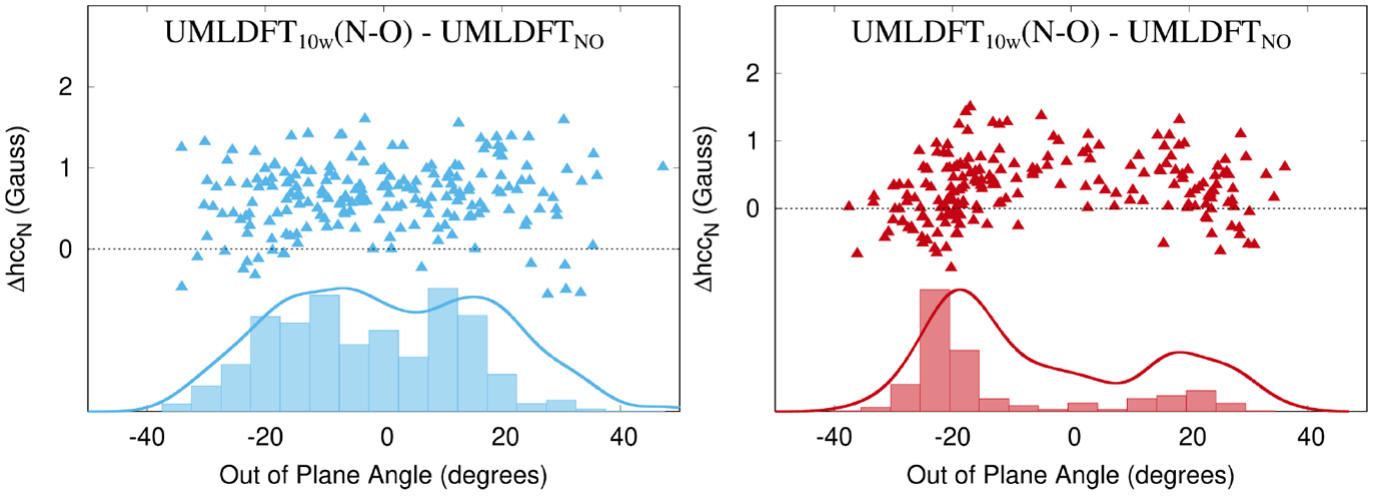
Computed UMLDFT_10w_(N–O)/FQ - UMLDFT_NO_ Δhcc_N_ (Gauss) as a function of the out-of-plane angle for PROXYL (left) and TEMPO (right) in an aqueous solution. Histograms and Gaussian convolutions of absolute Δhcc_N_ values are also given.

The results reported in the previous sections assume active and inactive regions to be noncovalently bonded. However, UMLDFT can in principle be applied to covalently bonded fragments. To demonstrate the method’s potentialities, the active space is reduced to the N–O group only, and the remaining atoms of the radicals are included in the inactive UMLDFT region, together with selected water molecules. This approach is denoted as UMLDFT_NO_/FQ, and computed hcc_N_’s are reported in Table 2. UMLDFT_NO_/FQ results differ of about 0.7 (PROXYL) and 0.3 (TEMPO) Gauss with respect to UMLDFT_10w_/FQ data, which can be taken as a reference. However, as already reported above, in order to better analyze the computed results, in Figure 5, UMLDFT_NO_/FQ-UMLDFT_10w_(N–O)/FQ differences as a function of the OOP angle are reported for PROXYL (left) and TEMPO (right). Absolute error distributions are also given as histograms, and their convolution with a Gaussian-type function is plotted.

Both PROXYL and TEMPO Δhcc_N_ distributions are comparable to those already commented on in Figure 4. However, in the present case, Δhcc_N_ values show very large variability, from −0.6 to 1.6 G, with a standard deviation of about 0.5 and 0.4 G for PROXYL and TEMPO, respectively. The UMLDFT is able to provide average hcc_N_ values in good agreement with reference results by only considering a minimal active portion. Such features may be particularly useful for the extension of the approach to correlated Hamiltonians, which can be exploited to describe the active fragment.

### Comparison with Experimental Data

We finally move to compare computed and experimental data (See Table 2.). It has been reported in previous studies that DFT has strong limits at reproducing hcc_N_ values, due to an inappropriate account of electron correlation.^72,79^ However, UMLDFT results are in good agreement with most accurate QM/MM results reported by some of us^72^ and which are labeled as “best QM/MM” in Table 2. Such data were obtained by describing water molecules at the FQ level, with the further (and substantial) inclusion of Pauli repulsion and dispersion contributions, as computed at the QM level.^10−12,72^ The discrepancy between UMLDFT and experimental data is similar to “best QM/MM” results (1.1 ± 0.2 G for PROXYL and 1.1 ± 0.2 G). It is worth remarking that the “best QM/MM” requires a suitable parametrization of electrostatic (polarization) and Pauli repulsion terms for the specific case of aqueous solutions. Although such a “best QM/MM” formulation is indeed general,^10^ to be efficiently applied to generic environments, parametrization is always needed. In contrast, UMLDFT resorts to a full QM treatment (at the DFT level) of both the target system and its surroundings. Therefore, its application to any kind of external environment is straightforward.

Finally note that a proper account of electron correlation, e.g. by resorting to correlated coupled cluster calculations, is expected to reduce the computed error.^72^ However, computed UMLDFT/FQ differences between PROXYL and TEMPO hcc_N_ values (0.9 G) are perfectly in agreement with experimental findings (0.9 G), thus demonstrating that our approach can reliably describe different radical species.

## 5 Summary, Conclusions, and Future Perspectives

In this work, we have introduced a novel class of multiscale QM/classical approaches aimed at describing the electronic properties of open shell systems. The methods are based on the coupling of multilevel HF/DFT, which are extended to the unrestricted formalism, with an outer region described at the classical MM level. Similar to MLHF and MLDFT, UML methodologies are based on a partition of the QM layer into an active and an inactive part. The partitioning is performed on the initial α and β density matrices through a partial Cholesky decomposition of the occupied MOs, and virtual orbitals are obtained by means of PAOs. Note that the active occupied MOs may be further refined by means of a localization procedure targeted to specific molecular regions assigned to the active fragment. To this purpose, the energy-based approach developed by some of us can be extended to the unrestricted formalism.^33^ The UML methods proposed here may substantially reduce the computational cost associated with common ab initio calculations, because only the active subsystem MOs enter the SCF procedure, whereas the inactive density matrix is kept fixed. To further reduce such a computational cost, linear scaling implementations recently proposed for the restricted formulation can be extended to the unrestricted case too.^32^ UMLHF/DFT are coupled to an outer MM layer described in terms of nonpolarizable or polarizable force fields. In this way, not only the computational cost is further reduced but also a correct physicochemical description of the main interactions is preserved. Indeed, the MM part allows for an effective modeling of long-range electrostatics (and polarization forces) in a multiscale fashion.

To test the quality of the approaches, they are applied to compute the hcc_N_’s of PROXYL and TEMPO radicals dissolved in an aqueous solution. First, the quality of the designed computational protocol is tested on a single snapshot, demonstrating the necessity of including long-range electrostatics and polarization effects by comparing UMLDFT/TIP3P and UMLDFT/FQ results. The results also show that, at least for the selected systems and property, the rather crude description of active-inactive polarization effects which is achieved at the second step of our computational protocol is sufficient to correctly reproduce full DFT results. However, to consistently include such terms, “freeze-and-thaw” cycles, similar to what has been proposed in the context of FDE, would be necessary.^80^ PROXYL and TEMPO hcc_N_’s are then calculated as an average on a set of uncorrelated snapshots extracted from classical MD runs, which allow to correctly take into account the dynamical aspect of the solvation phenomenon. The computed data are perfectly in agreement with the best computational estimates proposed in the literature for the same systems and correctly reproduce the experimental findings, thus demonstrating the reliability of the developed methods for real-case systems. Also, we showcase the flexibility of UMLDFT/MM partitioning by including the nitroxyl group only in the active part, i.e. by considering covalently bonded fragments. Although in this case computed hcc_N_’s are not perfectly in agreement with our best estimates, such flexibility paves the way to the extension of a coupled cluster treatment of the active part, in order to take into account electron correlation, which may largely affect the electronic properties of open shell systems.^72,79^

On the other hand, the developed approach can also be extended to treat linear response properties of open shell systems by means of time-dependent DFT (TD-DFT) formulations. Also, UMLDFT/MM is tested here on aqueous solutions; however, the model is general enough to be applied to different solvents^16^ or different embedding environments, such as biological matrices or nanostructured materials. Finally, UMLDFT may also be coupled to more sophisticated polarizable force fields, which improve the description of specific anisotropic interactions.^64^

## Supporting Information Available

PROXYL hcc_N_ calculated at UMLDFT_*n*w_(/TIP3P,/FQ) levels and associated graphical representation for PROXYL (PDF)
Molecular geometries of PROXYL and TEMPO in aqueous solution exploited in this work (ZIP)
