# Supplementary material for: Integrated Multiscale Multilevel Approach to Open Shell Molecular Systems
Source: J Chem Theory Comput. 2023 Feb 13;19(5):1446–56. doi: 10.1021/acs.jctc.2c00805 (PMC10018740; doi:10.1021/acs.jctc.2c00805)
Supplement: Supplementary file 1 — ct2c00805_si_001.pdf [file ct2c00805_si_001.pdf]

Supporting Information: Integrated Multiscale Multilevel Approach to Open Shell Molecular Systems

Tommaso Giovannini,*,† Gioia Marrazzini,† Marco Scavino,† Henrik Koch,†,‡ and Chiara Cappelli*,†

†*Scuola Normale Superiore, Piazza dei Cavalieri 7, 56126 Pisa, Italy.*

‡*Department of Chemistry, Norwegian University of Science and Technology, 7491 Trondheim, Norway*

E-mail: tommaso.giovannini@sns.it; chiara.cappelli@sns.it

Table S1: Calculated UMLDFT$_{nw}$(/TIP3P; /FQ) hcc$_{\mathrm{N}}$ for a randomly selected snapshot of PROXYL, with different selections of active water molecules.

		PBE0/N07D			PBE0/N07D/6-31G(w)		
	UMLDFT$_{nw}$	Gas-phase	FQ	TIP3P	Gas-phase	FQ	TIP3P
	0w	13.2	14.1	13.8	13.3	14.2	13.9
**N-O**	2w	13.1	14.1	13.7	13.2	14.1	13.8
	5w	13.0	14.0	13.6	13.2	14.1	13.7
	10w	13.2	14.2	13.8	13.3	14.2	13.8
**CM**	2w	13.1	14.1	13.7	13.3	14.2	13.8
	5w	13.1	14.1	13.7	13.2	14.1	13.8
	10w	13.0	14.0	13.7	13.2	14.1	13.7
	Full DFT (3.5Å)	13.2	14.1	13.8	13.2	14.1	13.8
	Full DFT (6.5Å)	–	–	–	–	14.0	–

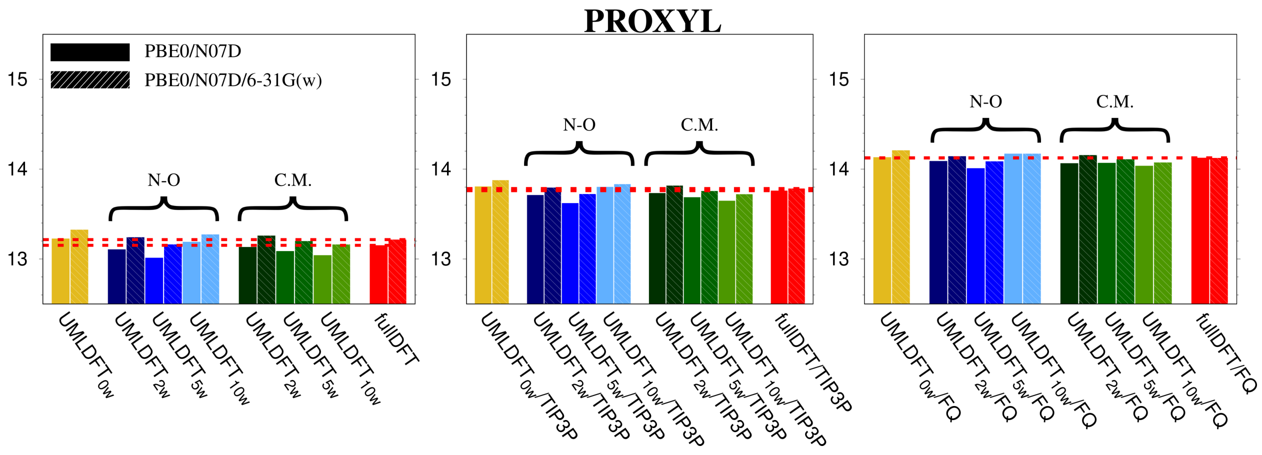

Figure S1: hcc$_{\mathrm{N}}$ (Gauss) calculated at the UMLDFT$_{nw}$(/TIP3P,/FQ) and full DFT(/TIP3P, /FQ) levels for a randomly selected snapshot of PROXYL. Water molecules included in the UMLDFT layer are selected with respect to the N-O group or C.M. Horizontal red lines correspond to full PBE0/N07D, PBE0/N07D/TIP3P and PBE0/N07D/FQ results.
